# Claims for disease-modifying therapy by Alberta non-insured health benefits clients

**DOI:** 10.1186/s12913-016-1685-y

**Published:** 2016-08-24

**Authors:** Cheryl Barnabe, Bonnie Healy, Andrew Portolesi, Gilaad G. Kaplan, Brenda Hemmelgarn, Charles Weaselhead

**Affiliations:** 1 Department of Medicine, Cumming School of Medicine, University of Calgary, 3330 Hospital Dr NW, Calgary, AB T2N 4N1 Canada; 2 Department of Community Health Sciences, Cumming School of Medicine, University of Calgary, 3280 Hospital Dr NW, Calgary, AB T2N 4Z6 Canada; 3 Alberta First Nations Information and Governance Centre, Suite 111, 535 8 Avenue SE, Calgary, AB T2G 5S9 Canada; 4 First Nations and Inuit Health Branch, Health Canada, Address Locator 0900C2, Ottawa, ON K1A 0K9 Canada; 5 Alberta First Nations Leadership Table, 101-12111 40 St SE, Calgary, AB T2Z 4E6 Canada

**Keywords:** Arthritis, Inflammatory bowel diseases, Psoriasis, Immunosuppressive agents, Antirheumatic agents, Pharmacoepidemiology

## Abstract

**Background:**

Uncontrolled disease activity in inflammatory diseases of the joints, skin and bowel leads to morbidity and disability. Disease-modifying therapies are widely used to suppress this disease activity, but cost-coverage is variable. For Treaty First Nations and Inuit people in Canada without alternative private or public health insurance, cost-coverage for disease-modifying therapy is provided through Non-Insured Health Benefits (NIHB). Our objective was to describe the prevalence and patterns of treatment with disease-modifying therapy for the NIHB claimant population, and also examine adjuvant therapy (analgesics, non-steroidal anti-inflammatory drugs (NSAIDs), corticosteroids) use.

**Methods:**

Cases (*n* = 2512) were defined by ≥1 claim for a disease-modifying anti-rheumatic drug (DMARD) or biologic between 1999 and 2012 in the NIHB pharmacy claim database. The proportion of the population with claims for individual agents and drug classes annually was calculated to estimate annual incidence and prevalence rates for use of disease-modifying therapy, and the prevalence of use of individual DMARDs, biologics and adjuvants. Differences in the proportion accessing adjuvant therapies and median doses in the 6 months following initiation of disease-modifying therapies was estimated.

**Results:**

The incidence rate of treatment was calculated at an average of 127.5 cases per 100,000 population between 2001 and 2012, and the cumulative prevalence, accounting for patients lost to the database, increased and then stabilized at 1.3 % in the last three years of the study. Annual dispensation of methotrexate, combination DMARD therapy and biologic therapy approached 35 %, 19 %, and 10 % of the cohort respectively. A declining prevalence of claims for acetaminophen (28 % to 15 %) and anti-inflammatories (73 % to 63 %) occurred from 2000 to 2012, however corticosteroid (32 %) and opioid (65 %) dispensation remained stable. The proportion of patients with claims for NSAIDs (69.9 % to 61.1 %, *p* = 0.002), oral corticosteroids (45.4 % to 33.6 %, *p* < 0.001) and parenteral corticosteroids (16.2 % to 8.3 %, *p* = 0.002) decreased in the 6 months following biologic initiation.

**Conclusions:**

The proportion of NIHB clients with active claims for disease-modifying therapy is lower than expected based on existing epidemiologic knowledge of the prevalence of inflammatory conditions in the First Nations and Inuit populations. These findings should be further explored in order to optimize treatment outcomes for NIHB claimants with inflammatory disease.

## Background

Inflammatory diseases of the joints (e.g. rheumatoid arthritis, spondyloarthropathies) [1], bowel (e.g. Crohn’s Disease, ulcerative colitis) [2–4] and skin (e.g. psoriasis) [5] are leading causes of disability in Canadian society. These chronic conditions are characterized by immune-mediated inflammation of their respective target organs, along with systemic consequences that result in premature death. Although these diseases seem diverse and can occur in isolation, their pathogeneses overlap [6, 7] and they are treated with the same medications. Disease-modifying anti-rheumatic drugs (DMARDs), such as methotrexate, sulfasalazine, or leflunomide, inhibit synthesis of deleterious inflammatory cytokines. Biologic therapies, including anti-tumor necrosis factor α agents, B-cell depletion and T-cell costimulation inhibition strategies, block the activation or consequences of inflammatory cascades that trigger clinical inflammation.

Epidemiology studies on the prevalence of inflammatory conditions in the Indigenous peoples in Canada highlight differences based on ancestry and disease. Rheumatoid arthritis affects twice as many First Nations persons as compared to non-First Nations [8], and the Haida population of British Columbia has seven times the rate of ankylosing spondylitis compared to the general population [9]. In contrast, inflammatory bowel diseases such as Crohn’s disease and ulcerative colitis are considered rare in First Nations populations [10], and rheumatoid arthritis was not more common in Inuit populations [11]. To date, no published studies have characterized psoriasis or psoriatic arthritis prevalence or phenotype in any Indigenous groups. Inflammatory arthritis among First Nations People is also more severe [12] and is associated with worse disability [13] compared to non-First Nations. Timely diagnosis and effective use of disease-modifying therapy are proven strategies to reduce the impact of disease, as summarized in Solomon et al. [14]. Beyond logistical barriers [15], disempowering experiences with the healthcare system [16] will decrease the likelihood of early diagnosis and frequent re-evaluation of disease activity needed to secure the best outcomes. An additional consideration is the ability to access medications, which is different for First Nations and Inuit patients with Treaty Status under the Indian Act compared to other Canadians, with the federal government being responsible for pharmaceutical drug cost-coverage through the Non-Insured Health Benefits (NIHB) program, if not otherwise available through private or provincial plans [17]. All approved prescribers can initiate and renew prescriptions for DMARDs and adjuvant therapy prescriptions for symptom relief (including acetaminophen, non-steroidal anti-inflammatory drugs (NSAIDs), opioids and corticosteroids). Biologic therapies are limited use benefits, with specific criteria for provision such as requiring the prescriber to be a medical specialist, and the patient having active disease despite the use of first-line therapies. The aim of our study was to examine prevalence of use with DMARDS and biologics for patients covered by the NIHB formulary by examining paid claims. We also sought to describe concomitant dispensation of adjuvant therapies. This information will contribute to the understanding of the quality of care provided to First Nations and Inuit patients requiring disease-modifying therapy.

## Methods

### Data source/elements

The NIHB Program, administered by the First Nations and Inuit Health Branch (FNIHB), provides financial coverage for formulary prescription medications for registered First Nations and recognized Inuit populations in Canada when not covered by another private or public drug plan. A recent estimate suggests that the national utilization rate for NIHB is estimated at 62 %. Each individual has a unique client identification number, and each claim for medication paid for by NIHB is recorded in the database. Medication class, therapeutic class, chemical name, and drug identification number, as well as dose and strength of the medication, the date of dispensation, and quantity dispensed are recorded. Alberta NIHB pharmacy claims only were analyzed in this study.

### Population denominator

A migration-adjusted total eligible claimant population residing in the province of Alberta is calculated on March 31 of each year and was provided by FNIHB.

### Case definition

Cases were defined by at least 1 claim for either a DMARD or Biologic at any time during the study period (January 1, 1999 to December 31, 2012). DMARDs included methotrexate, sulfasalazine, hydroxychloroquine, azathioprine, cyclosporine, gold, and leflunomide, whereas biologics included anakinra, infliximab, adalimumab, etanercept, golimumab, certolizumab pegol, rituximab, tocilizumab, and abatacept. To increase the specificity of the case definition by identifying claimants most likely to have inflammatory joint, skin or bowel disease, but limited by restricted ability to link to primary records or administrative data, we excluded individuals who also received medications primarily indicated to prevent transplant organ rejection (tacrolimus, sirolimus, mycophenolate mofetil).

### Claims analyzed

For individuals meeting the case definition, all DMARD, biologic, analgesic (acetaminophen, opioids), NSAIDs and corticosteroids (oral, injection) claims during the study period were available for analysis.

### Incident cases

We applied a 2 year run-in period to define incident cases, such that those individuals with claims for a DMARD or biologic in fiscal years 1999/2000 and 2000/2001 were considered to have prevalent disease, and those individuals with no claims meeting the case definition in those years but subsequently meeting the case definition were considered incident in the year of the first claim.

### Outcomes and statistical analysis

SAS (version 9.3) was used to analyze the following outcomes:

- *i) Incidence and prevalence rates for treatment:* The cumulative prevalence rate for treatment, reported as cases per 100 population, included individuals who met the case definition and continued to have active claims for a DMARD or biologic agent in each fiscal year; cases were removed from the numerator if a fiscal year lapsed with no claims for any of the agents of interest. The incidence rate, reported as new cases under treatment per 100,000 population, included individuals meeting the case definition for the first time in each fiscal year excluding the run-in period. The denominator for both the cumulative prevalence and the incidence rate was the migration-adjusted population total registered in the province on March 31 of that year, thus accounting for patients lost to the database.
- *ii) Proportional distribution of use of DMARDs and/or biologics in prevalent cases:* In clients under active treatment, the annual prevalence of drug use was calculated for DMARDs and/or biologics as therapeutic classes, as well as for individual agents in these classes, by examining paid claims. The number of individuals with at least one claim in each year of the database for the agent of interest was used as the numerator, and the denominator was the cohort of patients meeting the case definition who remained prevalent in the database.
- *iii) Prevalence of use of adjuvant therapies:* The proportion of the prevalent cases with claims for acetaminophen, opioids, NSAIDs and/or corticosteroids was calculated in each year of the database.
- *iv) Use of adjuvants prior to and following DMARD or biologic initiation:* To determine the effect of disease-modifying therapy initiation on the need for adjuvant therapy, we compared the prevalence of claims and median daily dose of adjuvant agents as a class in the six months prior to the prescription for a DMARD or biologic, to a six month time period following the dispensation of the first DMARD or biologic. For the patients exclusively treated with DMARDs, we included cases with a first claim for any therapeutic agent after April 1, 2002. All the patients treated with biologic therapies were considered incident cases and could be included in this analysis, as the study period included years prior to the availability of biologic therapies. McNemar’s test was used to test for changes in the prevalence of use during the two time periods, whereas the comparison of median daily doses was by Wilcoxon signed rank test. Median doses were calculated from the dose of the medication, number of doses supplied, and number of days supplied, applying conversions of opioids to an oral morphine equivalent (http://nationalpaincentre.mcmaster.ca/opioid/cgop_b_app_b08.html), steroids to an oral prednisone equivalent (http://www.globalrph.com/corticocalc.htm), and NSAIDs to an ibuprofen equivalent (http://www.ncbi.nlm.nih.gov/books/NBK65641/).

## Results

### Cohort of patients with inflammatory disease

A total of 2578 individuals received at least 1 claim for a DMARD or biologic during the study period. After applying exclusion criteria, 2512 individuals remained for analysis, including 1853 females (73.8 %). A total of 217 individuals (8.6 %) entered the database at <18 years of age, and most were under the age of 60 years at the date of the first recorded claim (*n* = 2330; 92.8 %). The majority of patients (*n* = 1658; 66.0 %) had their first claim in the database in 1999, for a median of 12.8 (IQR 8.3–13.8) years of follow-up per person. The median number of claims for the drugs of interest was 94.0 (IQR 30.0–243.0) per individual, and a total of 442,082 claims were analyzed.

**Fig. 1:**
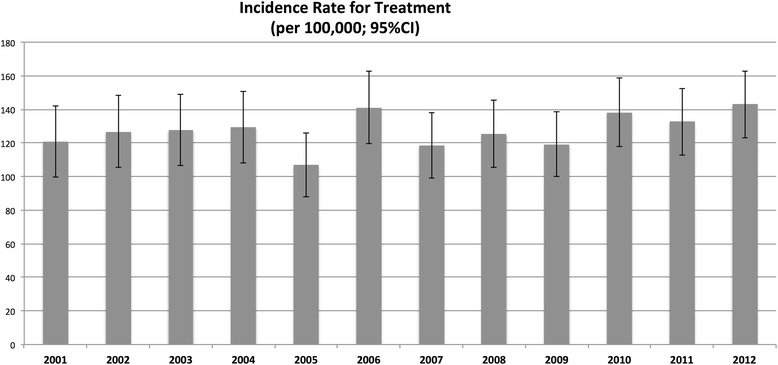
Incidence rate of initiation of DMARDs or biologic therapies in Alberta non-insured health benefits clients

**Fig. 2:**
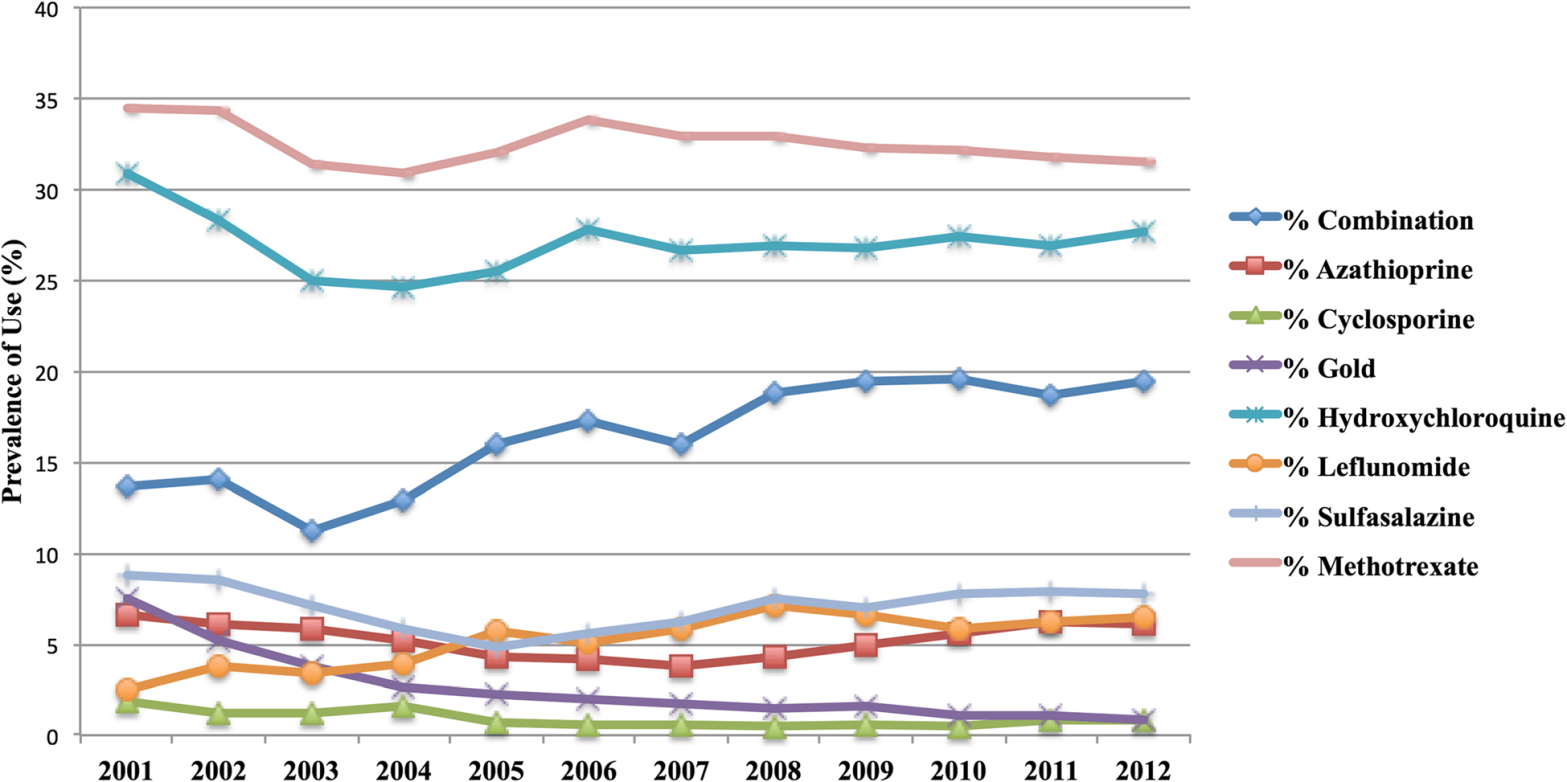
Prevalence of DMARD use by agent, 2001–2012

**Fig. 3:**
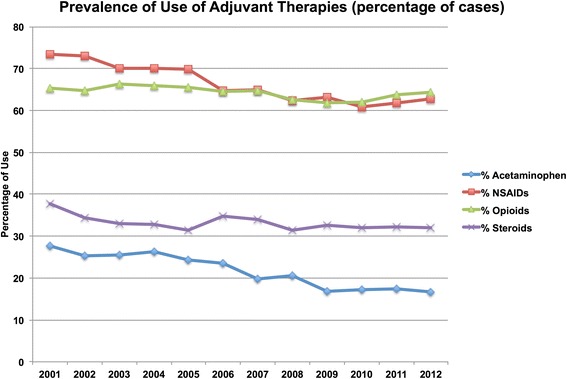
Prevalence of use of adjuvant therapies

- *i) Incidence and prevalence rates for treatment:* In 1999, a total of 318 individuals had a claim for a DMARD, increasing to 2512 ever-claimed during the study period and with 1725 prevalent cases in 2012. The incidence rate of treatment was calculated at an average of 127.5 cases per 100,000 population between 2001 and 2012, with a peak in 2012 at a rate of 143.1 cases per 100,000 population (Fig. 1). During this same time the cumulative prevalence, accounting for patients lost to the database, increased and then stabilized at 1.3 % in the last three years of the study.
- *ii) Proportional distribution of use of DMARDs and/or biologics in prevalent cases:* The proportion of patients meeting the case definition and receiving at least one claim in the fiscal year for individual DMARD agents is depicted in Fig. 2. Methotrexate was used by 30 to 35 % of cases in each year, and combination DMARD therapy by 11 to 19 % of cases in each year. The use of sulfasalazine, azathioprine and leflunomide remained below 10 %, with negligible use of cyclosporine and gold by 2012.
  The first claim for a biologic agent was in 2002, with a total of 266 individual users during the study period. The prevalence of use in 2012 was 9.8 % (*n* = 169/1725). The majority of biologics were used in combination with DMARDs (ranging from 54 to 76 % in each year). Etanercept was the most frequently claimed biologic during the study period (*n* = 815/1149 claims; 70.9 %), followed by adalimumab (*n* = 204/1149 claims; 17.8 %).
- *iii) Prevalence of use of adjuvant therapies:* (Fig. 3)*:* A declining annual prevalence of claims for acetaminophen (from 27.7 % in 2001 to 14.8 % in 2012), and NSAIDs (from 73.3 % in 2001 to 62.9 % in 2012) was observed. The proportion of patients with corticosteroid and opioid claims was stable, with an annual range of 31.3–37.7 % and 61.9–66.3 % of patients respectively.
- *iv) Use of adjuvants prior to and following DMARD or biologic initiation:* A total of 456 individuals had their first service date after 2002 and had claims for DMARDs only, forming the cohort of incident DMARD-treated cases. The prevalence of use of acetaminophen, NSAIDs, opioids and corticosteroids in the 6 months prior to and following DMARD initiation these cases is shown in Table 1. The use of oral corticosteroids increased in this incident cohort in the 6 months following DMARD initiation compared to the 6 months before, rising from 21.9 % to 31.1 % (*p* < 0.001). Median doses of NSAIDs dispensed were observed to increase as well (Table 2).

**Table 1:** Prevalence of use of adjuvant therapies for incident cases receiving DMARDs and individuals receiving biologics, 6 months pre-post

	Incident DMARD users (*n* = 456)			Biologic users (*n* = 229)^a^		
	6 months prior to DMARD start	6 months following DMARD start	*p* value	6 months prior to biologic start	6 months following biologic start	*p* value
Acetaminophen	21 (4.6 %)	21 (4.6 %)	1.0000	29 (12.7 %)	27 (11.8 %)	0.8
NSAIDs	162 (35.5 %)	165 (36.2 %)	0.8682	160 (69.9 %)	140 (61.1 %)	0.002
Opioids	95 (20.8 %)	113 (24.8 %)	0.0918	154 (67.3 %)	147 (64.2 %)	0.2
Oral Steroids	100 (21.9 %)	142 (31.1 %)	0.0001	104 (45.4 %)	77 (33.6 %)	<0.001
Parenteral Steroids	13 (2.9 %)	22 (4.8 %)	0.1221	37 (16.2 %)	19 (8.3 %)	0.002

^a^Includes patients with data available for 6 months prior to and following biologic initiation

**Table 2:** Comparison of median doses (interquartile range) in the six months prior to and six months after inflammatory disease therapy initiation

	Acetaminophen	NSAIDs^a^	Opioids^b^	Corticosteroids^c^
DMARDs Only (Incident to database after 2002) *n* = 456	Pre: 180.6 mg (88.9–500) Post: 277.8 mg (88.9–666.7) (*p* = 0.7)	Pre: 455.6 mg (216.7–866.7) Post: 547.9 mg (233.3–1067.7) (*p* = 0.02)	Pre: 3.3 mg (1.1–9.4) Post: 3.4 mg (0.8–14.0) (*p* = 0.08)	Pre: 6.2 mg (1.8–11.4) Post: 5.3 mg (2.1–13.7) (*p* = 0.7)
Biologic Users^d^ *n* = 229	Pre: 277.8 mg (166.7–833.3) Post: 277.8 mg (83.3–555.6) (*p* = 0.5)	Pre: 600.0 mg (305.3–1200.0) Post: 633.3 mg (300.0–1333.3) (*p* = 0.2)	Pre: 18.0 mg (5.0–56.1) Post: 22.3 mg (7.4–58.4) (*p* = 0.04)	Pre: 5.0 mg (1.75–10.0) Post: 5.8 mg (2.1–10.6) (*p* = 0.02)

^a^mg of ibuprofen equivalent

^b^mg of morphine equivalent

^c^mg of prednisone equivalent

^d^Includes patients with data available for 6 months prior to and following biologic initiation

### Biologics

Patients initiated on biologic therapy were prescribed a median of 3 DMARDs before the biologic started. In the six months after starting a biologic, the median number of DMARDs maintained was 1 (*p* < 0.001, Wilcoxon sign rank test). Furthermore, whereas 24 individuals did not have a claim for a DMARD in the 6 months prior to biologic start, this increased to 56 individuals in the 6 months following a biologic start. A significant reduction in the prevalence of use of NSAIDs and corticosteroids was seen in the 6 months following the initiation of biologic therapy. The proportion of cases with claims for NSAIDs decreased from 69.9 % to 61.1 % (*p* = 0.002), as did the proportion with claims for oral corticosteroids (45.4 % to 33.6 %, *p* < 0.001) and parenteral corticosteroids (16.2 % to 8.3 %, *p* = 0.002). However there was no difference in the proportion of cases with claims for acetaminophen and opioids (Table 1). Median doses of opioids were observed to increase, as did the median dose of corticosteroids for those continuing to use them (Table 2).

## Discussion

Our analysis presents data on the prevalence of accessing medication cost-coverage for disease-modifying therapies and adjuvant therapies by Treaty First Nations and Inuit peoples in Alberta through the Non-Insured Health Benefits Plan. Annually, approximately 1.3 % of the population is accessing disease-modifying therapy through this program. When placed in the context of underlying inflammatory disease prevalence, for which disease-modifying therapy is indicated, this leads us to believe that it is likely that many First Nations and Inuit in Alberta are not receiving pharmacotherapy for their conditions. Epidemiology studies have estimated that rheumatoid arthritis alone has a prevalence of nearly 1 % in the general Canadian population [1], but is twice as high in the predominantly Algonquin First Nations population of Manitoba [8], and even higher in other tribal areas of North America [18, 19]. In Alberta, our estimate of the prevalence of rheumatoid arthritis in First Nations people is 3.2 per 100 population, with the ankylosing spondylitis rate at 0.6 per 100 population, and the psoriatic disease (skin and/or arthritis) rate at 0.3 per 100 population [20]. Thus, while NIHB clients with inflammatory disease are presenting for treatment as seen in the incidence rate for treatment in this study, they are not being retained in care, reflected in the low prevalence of claims for DMARDs and biologics annually in relation to inflammatory disease prevalence rates.

Our study also provides information on annual use of individual DMARDs and biologic agents. Methotrexate claims were made by 30 to 35 %, and biologic therapy claims by 10 % of cases in our study annually. No direct comparison of rates to the general population requiring disease-modifying therapy is available. However, we expect that our data largely reflects patients with rheumatoid arthritis, due to it being the most common inflammatory disorder for which DMARDs and biologics are used. Methotrexate is the first-line therapy indicated for rheumatoid arthritis, and was used by 72 % of patients in a recently reported Canadian cross-sectional study [21]; estimates of rates of use of biologics for rheumatoid arthritis from population-based administrative datasets in Ontario was 23.5 % [22], similar to that in the Netherlands (22 %) [23], or the United States (26 %) [24]. Thus, our findings are counterintuitive given findings of worse arthritis severity in First Nations people [12]. A reassuring trend was the increased use of combination therapies in recent years. By 2012, approximately 20 % of DMARDs were used in combination, and the majority of patients with claims for biologic therapies concomitantly have claims for at least one DMARD, which improves efficacy and survival of biologic agents. Prior to our study, the only publication discussing therapeutic exposures for DMARDs was for Aboriginal patients with rheumatoid arthritis in Manitoba, who had more DMARD, combination therapy and corticosteroid exposures [12]. As the dataset used in our study does not allow exploration of reasons for our observations at the individual level, we cannot identify specifically what factors influence this perceived under-utilization of evidence-based therapies. We can only hypothesize potential explanations, such as medical contraindications to these agents, perceived difficulties in monitoring for adverse events, or patient concerns of toxicity. In addition to patient-specific reasons for not proceeding with biologic therapy, an additional consideration is that the low prevalence of use may reflect both difficulties in accessing specialist care, a necessary criteria of the NIHB formulary for a biologic prescription to be approved, as well as a multi-step process for limited use prescription approvals and renewals. FNIHB and the Canadian Rheumatology Association Optimal Care Committee have been working collaboratively to streamline the clinical information requested from medical specialists, and improve communication processes to ensure the physicians are aware of the status of claims. As well, changes in the NIHB formulary criteria effective March 2014 brings the required DMARD trials before biologics for rheumatoid arthritis patients in line with the current evidence-base. We encourage further activities that eliminate process inefficiencies and facilitate access to necessary therapies that may be barriers to achieving optimal disease outcomes for not only NIHB clients but all patients with inflammatory disease.

The study of the use of adjuvant therapy contributes to the understanding of management of symptoms of inflammatory diseases for which disease-modifying therapy is initiated. Although steroids are appropriate to manage severe disease or flares for limited time periods, their ongoing use and increasing dose indicates suboptimally treated disease, and places individuals at risk for complications of diabetes, hypertension, cardiovascular disease, infection, osteoporosis, glaucoma and cataracts. We found that approximately one-third of NIHB clients meeting the case definition were exposed to steroids during their disease course. Although opioids may be prescribed for other medical conditions, a high prevalence of use suggests under-treatment of the primary condition and/or permanent joint damage from untreated inflammation. Here, our results on the frequency of use of adjuvant therapies once a biologic was initiated are helpful in demonstrating the benefit of optimal treatment, as significant reductions in the proportion of clients using anti-inflammatories and both oral and parenteral corticosteroids occurred. This is however tempered by the finding of increases in the median doses of steroids and opioids in those continuing to receive these therapies, likely reflecting clients with more severe or refractory disease having persistent adjuvant therapy requirements. Our treatment goals should continue to focus on optimal treatment of inflammatory disease to reduce potential complications arising from exposure to these particular adjuvants.

Our study provides important information on treatment patterns and has advantages in being population-based, however we do acknowledge that the main limitation is the lack of clinical data to confirm diagnosis and provide information on disease severity or complications. We also assumed that corticosteroids were used for disease flares, although they may have been indicated for other conditions, such as exacerbations of chronic obstructive pulmonary disease or allergic reactions, and the entire therapeutic history of individuals was not analyzed. Population migration and delays in reporting of births and deaths may affect our estimates, but the magnitude of this error will be consistent throughout each annual time period examined. As is expected in pharmacotherapy studies, we were unable to confirm if the medication was actually taken by the patient, and the dataset excludes medications received in hospital, bought by individuals as over-the-counter therapies, or through private/provincial payment sources. We could not account for these nor claims made in other provinces in our exposure-duration assessment. Individual choices to not proceed with therapeutic recommendations may also factor in the observed patterns of use. Further research would be necessary to confirm our findings at the individual level and explore why the trends were observed. Finally, we were unable to stratify our analysis for First Nations and Inuit populations separately, although a low proportion of the total Alberta population is Inuit (<0.1 %) and we believe our data largely represents NIHB clients of First Nations descent. Separate studies to discover unique trends that may exist for Inuit populations should be considered.

## Conclusions

In summary, the proportion of NIHB clients with active claims for disease-modifying therapy is lower than anticipated, with low prevalence of use of biologic therapies. The rationale of these findings should be further explored in order to increase the use of effective therapies. In addition, health systems and providers need to re-evaluate how they can provide high quality care and services that actively engage Indigenous patients afflicted with inflammatory disease in care. The combination of changes in health system policies and healthcare delivery offer the opportunity to improve inflammatory disease outcomes for Indigenous patients in Canada.

## Abbreviations

DMARD: Disease modifying anti-rheumatic drug
FNIHB: First Nations and Inuit Health Branch
NIHB: Non-Insured Health Benefits
NSAIDs: Non-steroidal anti-inflammatory drugs
